# Digital engagement enhances dual GIP/GLP‐1 receptor agonist and GLP‐1 receptor agonist efficacy: A retrospective cohort analysis of a digital weight loss service on outcomes and safety

**DOI:** 10.1111/dom.70244

**Published:** 2025-10-27

**Authors:** Hans Johnson, Ashley Kieran Clift, Daniel Reisel, David R. Huang

**Affiliations:** ^1^ Department of Digital Health and Care University of Bristol Bristol UK; ^2^ Department of Education University of Oxford Oxford UK; ^3^ Department of Clinical Research Voy, Menwell Ltd London UK; ^4^ Department of Surgery & Cancer Imperial College London London UK; ^5^ EGA Institute for Women's Health University College London London UK

**Keywords:** digital health, GIP/GLP‐1 RA, GLP‐1 RA, obesity, retrospective study, safety, therapy, weight loss

## Abstract

**Aims:**

We evaluated the weight loss efficacy and safety of a national digital weight loss service (DWLS) and explored associations between digital engagement and outcomes in adults prescribed dual GIP/GLP‐1RA and GLP‐1RA.

**Materials and Methods:**

We conducted a retrospective longitudinal cohort analysis of adults prescribed dual GIP/GLP‐1RA and GLP‐1RA between August 2024 and July 2025 within the Voy DWLS in the United Kingdom (UK). Digitally engaged patients met all three criteria: (i) ≥1 coaching session; (ii) weekly weight logging (≥4/month); and (iii) ≥1 additional in‐app interaction. Weight‐loss trajectories were modelled using mixed models for repeated measures (MMRM). Kaplan–Meier methods and Cox models examined time to attain weight loss thresholds (≥5, ≥10, ≥15, ≥20, ≥25%). Safety events were summarised as rates per 1000 patient‐months with 95% CIs.

**Results:**

The cohort included 106 653 adults (mean age 42.3 ± 12.7 years; 77.9% female; baseline BMI 35.2 ± 6.2 kg/m^2^). Whilst 79.7% (*n* = 84 955) used the digital application, 5.7% (*n* = 6086) met maximal engagement, with 100 567 classed as not engaged. Across 11 months, engaged patients achieved greater adjusted weight loss than not engaged (21.5% [95% CI −22.0 to −21.1] vs. 17.0% [−17.2 to −16.8]; absolute difference 4.5 percentage points; *p* < 0.001). Kaplan–Meier analyses showed consistently higher likelihood of milestone attainment for engaged participants (≥5%: HR 1.42, 95% CI 1.38–1.46; ≥10%: HR 1.46, 1.41–1.52; ≥15%: HR 1.53, 1.45–1.61; ≥20%: HR 1.62, 1.50–1.75; ≥25%: HR 1.86, 1.64–2.10; all *p* < 0.001). Safety analyses over 290 050 patient‐months showed incidence 1.57 per 1000 patient‐months, with no excess risk in engaged groups (IRR 0.83, 95% CI 0.60–1.15).

**Conclusions:**

Digital engagement was associated with 4.5 percentage points greater weight loss (21.5% vs. 17.0%), faster milestone achievement, and comparable safety profiles.

## 1 INTRODUCTION

The obesity epidemic is one of the most pressing global challenges, where 38% of the global population is currently either overweight or obese, with predicted prevalence to reach 51% of the world population by 2035.^1^ Obesity is associated with a cascade of pathophysiological changes and interactions leading to a multitude of interconnected and consequential comorbidities, thereby substantially contributing to sequelae including cardiovascular disease, type 2 diabetes, cancer and even premature mortality.^2^

Traditional approaches to obesity management, such as lifestyle modifications alone have delivered limited long‐term efficacy, with individuals regaining the lost weight within as soon as 36 weeks post‐intervention, as illustrated by Machado and colleagues' meta‐analysis of 27 studies.^3^ The emergence of glucagon‐like‐peptide‐1 receptor agonists (GLP‐1RAs) and dual glucose‐dependent insulinotropic polypeptide (GIP)/GLP‐1 receptor agonists has fundamentally transformed obesity management, offering highly efficacious pharmacological approaches to weight loss. Recent meta‐analyses of randomised controlled trials (RCTs) demonstrate that tirzepatide achieves superior weight loss compared to placebo, with a mean difference of −16.32% (95% CI: −18.35 to −14.29).^4^

The latest landmark trials of GLP‐1 receptor agonists and dual GIP/GLP‐1 receptor agonists, such as semaglutide and tirzepatide, have shown substantial weight‐loss outcomes. In the SELECT trial, participants treated with semaglutide achieved a sustained mean weight loss of 10.2% at 208 weeks,^5^ while in the SURMOUNT‐5 trial tirzepatide led to a 20.2% weight loss at 72 weeks.^6^ Aronne Louis and colleagues demonstrated that both tirzepatide and semaglutide reduce waist circumference, systolic blood pressure, glycated haemoglobin, and lipid levels, in line with earlier RCTs.^7^, ^8^ However, RCT populations do not reflect real‐world effectiveness, whereas various factors including treatment adherence, lifestyle support, and healthcare engagement can significantly influence treatment outcomes.^9^, ^10^ Moreover, the integration of digital health technologies with pharmacotherapy represents an emerging approach that can enhance treatment effectiveness through personalised coaching, tailored monitoring, behavioural support encompassed around digital engagement.^11^, ^12^

Digital weight loss services (DWLS) have evolved rapidly, leveraging smartphone applications, remote coaching, and data analytics to provide scalable, personalised interventions.^13^ A meta‐analysis suggested that digital interventions can enhance weight‐loss outcomes when combined with standard approaches, although most evidence derives from studies conducted without concurrent dual GIP/GLP‐1RA or GLP‐1RA therapy, where clinically significant but modest weight loss was observed.^14^ The potential synergy between digital engagement and dual GIP/GLP‐1RA and GLP‐1RA therapies remains largely unexplored, despite theoretical benefits including improved medication adherence, enhanced lifestyle modification, and dynamic behavioural reinforcement.^15^

With the novelty of dual GIP/GLP‐1RA and GLP‐1RA and the potential for rapid patient onboarding at a population level, safety considerations are paramount when implementing the DWLS. While clinical studies such as the SURMOUNT and STEP trials have established the safety profiles of these medications,^6^, ^16^, ^17^ real‐world safety data, particularly in the context of digital health integration, remain limited. Understanding both efficacy and safety outcomes in large, diverse populations is essential for optimising patient care, equity of health and informing clinical practice guidelines.

In this study, we address these knowledge gaps through our primary objective where we sought to characterise real‐world WL efficacy, quantify the impact of digital engagement on outcomes, and establish the safety profile of integrated digital‐pharmacological obesity management with dual GIP/GLP‐1RA and GLP‐1RA over 11 months of follow‐up in the ‘Voy’ DWLS.

## 2 METHODS

### 2.1 Study design and participants

We conducted a retrospective cohort study of adults enrolled within the Voy DWLS across the United Kingdom (UK) between August 2024 and July 2025. Patients commenced medication at any point during this period (open cohort), resulting in variable follow‐up durations (1–11 months) and declining sample sizes at later timepoints due to both enrollment timing and treatment discontinuation. This comprehensive service delivers integrated obesity management by combining dual GIP/GLP‐1RA and GLP‐1RA prescribing alongside optional digital support modalities encompassing personalised coaching, weight monitoring applications, and educational resources. The investigation was devised as a service evaluation employing retrospective examination of routinely collected clinical data.

Participants comprised adults aged ≥18 years presenting with baseline body mass index (BMI) ≥30 kg/m^2^, or BMI ≥27 kg/m^2^ accompanied by at least one obesity‐associated comorbidity, who received dual GIP/GLP‐1RA and GLP‐1RA therapy (either semaglutide or tirzepatide, respectively) via the Voy platform (trading designation of Menwell Ltd) DWLS, a structured clinical weight management programme. Follow‐up was from the date of first dual GIP/GLP‐1RA and GLP‐1RA prescription until the earliest of treatment cessation (defined as discontinuation or target weight achievement) or reaching the end of the cohort window (censoring). As an integral component of their enrolment within the Voy DWLS, patients initiating pharmacotherapy could utilise the Voy application (app), through which they could schedule coaching consultations, monitor their weight trajectories, and interact with educational content. Each of these non‐pharmacological elements remained discretionary, accessed according to individual patient preference, and incurred no supplementary financial burden.

### 2.2 Outcomes and comparators definitions

The study utilised routinely available clinical data from the Voy app, such as body weight measurements (kg), height (cm), BMI (kg/m^2^) and baseline self‐reported comorbidities pertaining to polycystic ovarian syndrome (PCOS), high cholesterol, hypertension, diabetes mellitus, and metabolic dysfunction‐associated steatotic liver disease (MASLD). The primary outcome was percentage weight change from baseline at each month of follow‐up, modelled over 11 months. Secondary outcomes included achievement of clinically significant weight loss thresholds (≥5%, ≥10%, ≥15%, ≥20%, and ≥25% total body weight loss) and time to achievement of these milestones.^18^ Safety outcomes available through routinely collected internal Voy quality assurance and clinical governance data included all reported adverse events, categorised by severity, causality, and clinical significance. Incident severity was classified using a five‐level harm scale: no harm, minor harm, near miss, moderate harm, and major harm. Clinical incident types were categorised as prescribing errors, medication side effects, other clinical issues, inappropriate clinical information, follow‐up issues, and allergic reactions. Incident management status was tracked through four stages: closed/resolved, under investigation, initial triage, and quality assurance review. Additional risk stratification categorised incidents as very low risk, low risk, moderate risk, or unspecified risk.

### 2.3 Digital engagement definition

Digital engagement (‘engaged’) was defined as a composite measure requiring all three components to be considered maximally engaged: (1) attendance at ≥1 personalised lifestyle coaching session delivered remotely, (2) weight tracking ≥4 times per month (approximately weekly), and (3) ≥1 additional app interaction (accessing educational materials, goal setting, or other features). We have used this definition in our previous work capturing engagement and efficacy of WL treatment.^11^ This definition was designed to capture meaningful, sustained engagement rather than minimal platform use. If an individual only engaged in one or two of the above three, they were categorised as ‘not engaged’.

### 2.4 Data processing

Individuals were excluded from the study cohort if they had biologically improbable anthropometrics as follows: a baseline BMI >100 kg/m^2^, a velocity of BMI change exceeding ±4 kg/m^2^ in successive months, height <1.3 or >2.2 ms, or recorded weight under 40 kg or over 300 kg. These were applied with reference to the wider WL clinical team and existing literature.^19^ For individuals providing multiple weight measurements per month, an average measurement in the one‐month period was used and we recorded the frequency of reported measurements per month.

### 2.5 Statistical analysis

Baseline characteristics were summarised using means (SD) for continuous variables and counts (%) for categorical variables. Weight change was defined as percentage change from baseline (negative = loss), that is, 0% compared to month 0. Longitudinal trajectories over months 0 to 11 were analysed using mixed‐models for repeated measures (MMRM) with fixed effects for engagement status, month, and their interaction, adjusting for baseline age, BMI, sex, and comorbidities (diabetes, high cholesterol, hypertension, PCOS, MASLD). The MMRM model is designed to adjust for key baseline covariates such as age, sex, BMI and comorbidities. Within‐subject residual covariance was specified as compound symmetry with Satterthwaite degrees of freedom; prespecified sensitivity fits with alternative structures (e.g., first‐order autoregressive covariance structure [AR1]) were considered if convergence or fit warranted, as executed in previous clinical studies for WL.^7^ Missing data for the primary analysis were handled implicitly by the MMRM under a missing at random (MAR) assumption; thus, no imputation was performed. From the fitted MMRM we obtained marginal (adjusted) mean predictions of percentage weight change at each month between engaged and not engaged with 95% confidence intervals (CIs). The primary outcome was percentage weight change from baseline to the end of follow‐up. Because the analytic dataset contained months 0 to11, the primary contrast was baseline to month 11. To examine whether baseline weight influenced weight loss outcomes, we performed additional MMRM analyses with centred baseline weight as a predictor alongside engagement, month, and their interactions. Pearson correlations between baseline weight and 11‐month weight loss were calculated by engagement group.

Time to achieving weight‐loss thresholds (≥5%, ≥10%, ≥15%, ≥20%, ≥25%) was analysed using Kaplan–Meier methods, with log‐rank tests used to compare curves between engaged and non‐engaged groups; hazard ratios (HRs) with 95% CIs were estimated using Cox proportional hazards models with engagement as the exposure. Furthermore, we analysed the cohort for any weight regain for individuals who have reached at least 1 month of treatment and if they reverted to their baseline weight or gained weight beyond initial month zero baseline.

Safety outcomes were summarised as (i) cumulative risk per 1000 patients (exact Clopper‐Pearson 95% CIs) and (ii) incidence rates per 1000 patient‐months (exact Poisson 95% CIs). Between‐group effects included risk ratio (RR), risk difference (RD), odds ratio (OR), and incidence‐rate ratio (IRR).

A power analysis determined that a minimum of 118 participants per engagement group would provide 80% power to detect a 15% difference in the proportion of participants achieving ≥10% weight loss at a 5% significance level.

The study followed STROBE guidelines for observational research reporting (see Data S1).^20^ All analyses were conducted using R statistical software version 4.3.0. Statistical significance was defined as *p* < 0.05 for two‐sided tests.

### 2.6 Ethical considerations

This retrospective cohort service evaluation study was an analysis of de‐identified data collected during the routine clinical care of adults treated by Voy. The study was approved by the University College London (UCL) Research Ethics Committee (REC reference: 2025‐0906‐775). Consent for the collection of data from individual patients during the course of their care was obtained with the express consent that their anonymised information could be used for research and for the purpose of service improvement.

## 3 RESULTS

### 3.1 Baseline characteristics

The study cohort comprised 106 653 adults prescribed GIP/GLP‐1 receptor agonists and enrolled in the Voy DWLS. Of these, 84 955 participants (79.7%) used the weight‐loss app, meaning the majority of the users had uptake with the digital aspect of the service. Within this group, 6086 participants (5.7% of the total cohort) were classified as maximally engaged (≥1 coaching session AND weight tracking ≥4 times per month AND ≥1 app interaction), while 100 567 participants (94.3%) were considered not maximally engaged. Altogether, the cohort contributed 290 050 person‐months of follow‐up. The cohort was predominantly female (77.9%) with a mean age of 42.3 years (SD 12.7) and baseline BMI of 35.2 kg/m^2^ (SD 6.2).

Engaged participants were typically marginally older (45.0 vs. 42.2 years), had higher baseline weight (101.7 vs. 97.9 kg) and BMI (36.3 vs. 35.1 kg/m^2^), as well as a higher prevalence of comorbidities including PCOS (8.6% vs. 5.9%), hypertension (14.7% vs. 9.9%), and high cholesterol (10.9% vs. 6.7%) (Table 1).

**TABLE 1:** Baseline summary characteristics of the study cohort—engaged versus not engaged.

Characteristic	Engaged	Not engaged	Overall
	*N* = 6086	*N* = 100 567	*N* = 106 653
Anthropometrics			
Weight, kg	101.7 (21.7)	97.9 (20.4)	98.1 (20.5)
Height, cm	167.1 (8.8)	166.7 (9.3)	166.8 (9.2)
BMI	36.3 (6.7)	35.1 (6.2)	35.2 (6.2)
Demographics			
Age, years	45.0 (12.1)	42.2 (12.7)	42.3 (12.7)
Age group			
18–24	117 (1.9%)	6228 (6.2%)	6345 (5.9%)
25–34	1274 (20.9%)	25 833 (25.7%)	27 107 (25.4%)
35–44	1734 (28.5%)	28 090 (27.9%)	29 824 (28.0%)
45–54	1469 (24.1%)	21 071 (21.0%)	22 540 (21.1%)
55+	1492 (24.5%)	19 342 (19.2%)	20 834 (19.5%)
Gender			
Female	4908 (80.6%)	78 133 (77.7%)	83 041 (77.9%)
Male	1178 (19.4%)	22 434 (22.3%)	23 612 (22.1%)
BMI category			
Obese	5554 (91.3%)	91 645 (91.2%)	97 199 (91.2%)
Overweight	530 (8.7%)	8803 (8.8%)	9333 (8.8%)
Comorbidities			
Has diabetes	222 (3.6%)	3047 (3.0%)	3269 (3.1%)
Has high cholesterol	663 (10.9%)	6746 (6.7%)	7409 (6.9%)
Has high blood pressure	897 (14.7%)	9908 (9.9%)	10 805 (10.1%)
Has PCOS	525 (8.6%)	5941 (5.9%)	6466 (6.1%)
Has MASLD	211 (3.5%)	1777 (1.8%)	1988 (1.9%)
Engagement			
Tracking ≥4 readings	6086 (100.0%)	12 857 (12.8%)	18 943 (17.8%)
Any coaching session	6086 (100.0%)	26 334 (26.2%)	32 420 (30.4%)
Uses WL app	6086 (100.0%)	78 869 (78.4%)	84 955 (79.7%)

*Note*: Baseline age and body mass index were fully recorded with no missing data. Values presented as mean (SD) for continuous variables and *n* (%) for categorical variables.

Abbreviations: BMI, body mass index; MASLD, metabolic dysfunction‐associated steatotic liver disease; PCOS polycystic ovary syndrome; WL, weight loss.

### 3.2 Primary efficacy outcomes: longitudinal weight loss trajectories

**FIGURE 1:**
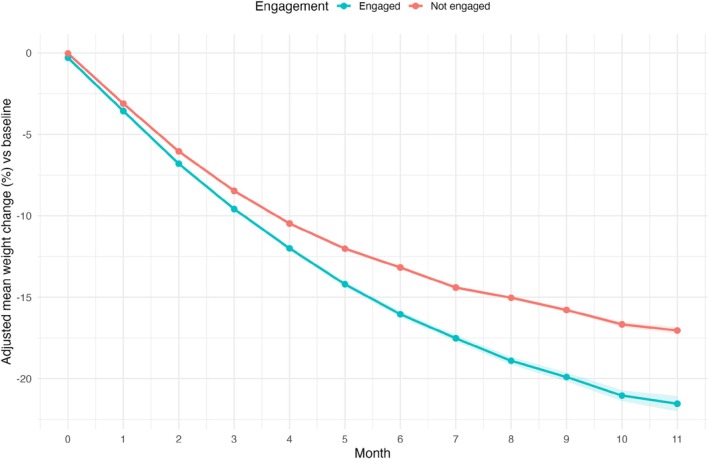
WL trajectories by digital engagement status over 11 months. MMRM‐derived adjusted mean percentage weight loss from baseline. Blue line: Engaged participants; pink line: Not engaged participants. Error bars: As shaded 95% CI. Model adjusted for baseline covariates.

MMRM analysis demonstrated progressive divergence in weight loss trajectories between engagement groups from month 1 onwards. At month 2, engaged participants achieved −6.8% (95% CI: −6.9, −6.7) weight loss compared with −6.0% (95% CI: −6.1, −5.9) in not engaged participants, representing a 0.8 percentage point difference favoring engaged participants (*p* < 0.001). This divergence amplified substantially by month 6, where engaged participants demonstrated −16.0% (95% CI: −16.2, −15.9) weight loss versus −13.2% (95% CI: −13.3, −13.1) in not engaged participants, yielding a 2.8 percentage point difference (*p* < 0.001), see Table 2 and Figure 1.

**TABLE 2:** Weight loss trajectories by engagement status from months 0 to 11 and number of participants engaged and not engaged throughout to follow‐up.

Month	(*N*) Engaged	(*N*) Not engaged	Engaged WL% (95% CI)	Not engaged WL% (95% CI)	Mean WL difference	Relative WL difference %	*p*‐value
0	6086	100 567	N/A	N/A	N/A	N/A	N/A
1	5839	46 254	−3.6 (−3.7, −3.4)	−3.1 (−3.2, −3.0)	−0.5	16.1	<0.001
2	5150	34 320	−6.8 (−6.9, −6.7)	−6.0 (−6.1, −5.9)	−0.8	13.3	<0.001
3	4070	24 997	−9.6 (−9.7, −9.5)	−8.5 (−8.6, −8.4)	−1.1	12.9	<0.001
4	2880	17 282	−12.0 (−12.1, −11.9)	−10.5 (−10.6, −10.4)	−1.5	14.3	<0.001
5	1936	12 465	−14.2 (−14.4, −14.0)	−12.0 (−12.1, −11.9)	−2.2	18.3	<0.001
6	1276	9470	−16.0 (−16.2, −15.9)	−13.2 (−13.3, −13.1)	−2.8	21.2	<0.001
7	843	6017	−17.5 (−17.7, −17.3)	−14.4 (−14.5, −14.3)	−3.1	21.5	<0.001
8	532	3948	−18.9 (−19.1, −18.7)	−15.0 (−15.1, −14.9)	−3.9	26.0	<0.001
9	387	2728	−19.9 (−20.2, −19.6)	−15.8 (−15.9, −15.7)	−4.1	26.0	<0.001
10	243	1548	−21.0 (−21.4, −20.7)	−16.7 (−16.8, −16.5)	−4.3	25.7	<0.001
11	120	786	−21.5 (−22.0, −21.1)	−17.0 (−17.2, −16.8)	−4.5	26.5	<0.001

*Note*: WL% = percentage weight loss from baseline (negative values indicate weight loss). Difference = engaged WL% − not engaged WL% (negative values favour engaged group for greater weight loss). *p*‐values derived from MMRM interaction terms (engagement × time). CI, 95% Confidence Interval; *N*, number of patients. All *p*‐values <0.001 indicate statistically significant differences between groups.

At the final 11‐month timepoint, engaged participants achieved −21.5% (95% CI: −22.0, −21.1) weight loss compared with −17.0% (95% CI: −17.2, −16.8) in not engaged participants, representing a maximum 4.5 percentage point difference (*p* < 0.001). All between‐group comparisons from month 1 through month 11 achieved statistical significance (*p* < 0.001).

Subgroup analysis by medication type revealed differential engagement effects between dual GIP/GLP‐1RA and GLP‐1RA monotherapy. At month 11, dual GIP/GLP‐1RA users achieved −22.0% (95% CI: −22.5, −21.5) weight loss when engaged versus −17.6% (95% CI: −17.8, −17.4) when not engaged, *p* < 0.001 (difference between groups: 4.4 percentage points). GLP‐1RA users demonstrated −19.0% (95% CI: −20.9, −17.1) weight loss when engaged versus −12.4% (95% CI: −13.2, −11.6) when not engaged, *p* < 0.001 (difference between groups: 6.6 percentage points). Both medication types showed superior outcomes with digital engagement, with GLP‐1RA users showing particularly strong benefit from engagement (see Table S1 [dual GIP/GLP‐1RA] and Table S2 [GLP‐1RA]). Both engaged cohorts of each medication type had 95% CI not overlapping, indicating a difference in efficacy between the medications.

### 3.3 Secondary efficacy outcomes: reaching weight loss thresholds Kaplan–Meier analyses, weight regain and influence of baseline weight

Kaplan–Meier survival analyses demonstrated a significantly higher likelihood of achieving clinically meaningful weight loss thresholds among engaged participants across all evaluated milestones (all log‐rank *p* < 0.001). For ≥5% weight loss, 79.4% of engaged participants achieved this threshold compared with 36.3% of not engaged participants by month 11 (hazard ratio [HR] 1.42, 95% CI: 1.38–1.46; relative risk 2.19). The advantage for engaged participants was more pronounced at higher thresholds: ≥10% weight loss was achieved by 50.6% vs. 20.5% (HR 1.46, 95% CI: 1.41–1.52; RR 2.47); ≥15% weight loss by 26.7% vs. 10.1% (HR 1.53, 95% CI: 1.45–1.61; RR 2.65); ≥20% weight loss by 12.2% vs. 4.3% (HR 1.62, 95% CI: 1.50–1.75; RR 2.84); and ≥ 25% weight loss by 4.9% vs. 1.5% (HR 1.86, 95% CI: 1.64–2.10; RR 3.34). The hazard ratios indicate that engaged participants had 42–86% faster time to achieving weight loss milestones, with the engagement advantage becoming more pronounced at higher weight loss thresholds (Table 3). The weight regain analyses showed that 1.1% (*N* = 66) of the engaged cohort experienced regain to baseline and 3.1% (*N* = 1612) of not engaged experienced regain to baseline weight. This demonstrates that digital engagement is associated with more sustained weight loss and lower regain rates.

**TABLE 3:** Weight loss milestone achievement by digital engagement status.

Weight loss threshold	Achievement (engaged group) %	Achievement (non‐engaged group) %	Hazard ratio for digital engagement(95% CI)	Relative risk	*p*‐value
≥5% weight loss	79.4%	36.3%	1.42 (1.38–1.46)	2.19	<0.001
≥10% weight loss	50.6%	20.5%	1.46 (1.41–1.52)	2.47	<0.001
≥15% weight loss	26.7%	10.1%	1.53 (1.45–1.61)	2.65	<0.001
≥20% weight loss	12.2%	4.3%	1.62 (1.50–1.75)	2.84	<0.001
≥25% weight loss	4.9%	1.5%	1.86 (1.64–2.10)	3.34	<0.001

*Note*: Achievement percentages represent the proportion of participants reaching each threshold by month 11. Hazard ratios compare engaged vs. not engaged participants. All comparisons are statistically significant by log‐rank test. CI, 95% Confidence Interval.

Higher baseline weight significantly predicted greater proportional weight loss across the intervention period (MMRM coefficient: β = 0.0093 per kg, 95% CI: 0.0085–0.0101, *p* < 0.001). This effect remained consistent across all monthly timepoints with no significant baseline weight × time interactions detected (all *p* > 0.05). Correlation analysis at 11 months revealed stronger associations between baseline weight and weight loss among engaged participants (*r* = −0.21, *p* = 0.021, *N* = 120) compared to not engaged participants (*r* = −0.11, *p* = 0.001, *N* = 786); negative correlation coefficients indicate that higher baseline weight predicted greater proportional loss.

### 3.4 Safety outcomes in dual GIP/GLP‐1RA and GLP‐1RA overall cohort population

Safety analysis identified 455 clinical incidents among 443 participants (4.15 per 1000 patients, 95% CI: 3.78–4.56) across 290 050 person‐months of exposure, yielding an incident rate of 1.57 per 1000 person‐months (95% CI: 1.43–1.72). Comparison of safety outcomes by engagement status revealed nuanced differences between groups.

Among engaged participants, 38 of 6086 (0.624%) experienced ≥1 incident compared with 405 of 100 567 (0.403%) not engaged participants, yielding a crude risk ratio (RR) of 1.55 (95% CI: 1.11–2.16) and a risk difference (RD) of 0.22% (95% CI: 0.02%–0.42%). However, when accounting for differential follow‐up duration, the incidence rate ratio (IRR) was 0.83 (95% CI: 0.60–1.15), that is, there was no significant difference in event rates per person‐time of exposure between engagement groups. The most common incident types were prescribing errors (*n* = 182; 40.0%; 1.71 per 1000 patients; 95% CI, 1.47–1.97) and medication side effects (*n* = 140; 30.8%; 1.31 per 1000 patients; 95% CI 1.10–1.55), with the majority classified as low‐level harm events: minor harm (*n* = 210, 46.2%; 1.97 per 1000 patients; 95% CI 1.71–2.25) and no harm (*n* = 191, 42%; 1.79 per 1000 patients; 95% CI 1.55–2.06). These findings suggest that while engaged participants may have a higher absolute risk of reported incidents, this likely reflects increased surveillance and reporting rather than a true increased risk when exposure time is considered (see Tables 4 and S1).

**TABLE 4:** Safety outcomes by digital engagement status.

Metric	Overall	Engaged	Not engaged	Effect measure (95% CI)
Participants, *n*	106 653	6086	100 567	N/A
Person‐months	290 050	29 409	260 641	N/A
Participants with ≥1 incident, *n* (%)	443 (0.42%)	38 (0.62%)	405 (0.40%)	RR: 1.55 (1.11–2.16)
Total incidents, *n*	455	39	416	RD: 0.22% (0.02%–0.42%)
Risk per 1000 patients (95% CI)	4.15 (3.78–4.56)	6.24 (4.42–8.56)	4.03 (3.65–4.44)	OR: 1.55 (1.11–2.17)
Rate per 1000 person‐months (95% CI)	1.57 (1.43–1.72)	1.33 (0.94–1.81)	1.60 (1.45–1.76)	IRR: 0.83 (0.60–1.15)

*Note*: Poisson confidence intervals were used for rates per 1000 patients and person‐months. Effect measures compare engaged vs. not engaged participants.

Abbreviations: CI, 95% confidence interval; IRR, incidence rate ratio; RD, risk difference; RR, risk ratio; OR, odds ratio.

## 4 DISCUSSION

In this large‐scale real‐world retrospective analysis of adults prescribed dual GIP/GLP‐1RA and GLP‐1RA within a national DWLS, we demonstrate that digital engagement confers substantial and clinically meaningful enhancement of WL efficacy. Digitally engaged participants achieved superior weight‐loss trajectories across all timepoints. By month 11, this culminated in a 4.5 percentage point absolute difference (−21.5% vs. −17.0%, *p* < 0.001). This corresponded to a 26.5% relative improvement attributable to digital engagement. The trajectories began to diverge progressively from month 2.

This engagement effect demonstrated striking consistency across clinically relevant thresholds, with engaged participants exhibiting 42%–86% faster time‐to‐achievement of milestone targets. Notably, 79.4% of engaged participants achieved ≥5% weight loss compared with 36.3% of non‐engaged participants (hazard ratio 1.42, 95% CI: 1.38–1.46), while more ambitious thresholds showed greater relative benefits (≥20% weight loss: 12.2% vs. 4.3%, hazard ratio 1.62, 95% CI [1.50–1.75]), which may potentially improve metabolic outcomes and reduce risk of co‐morbidities.^21^ These findings provide compelling evidence for a dose–response relationship between digital engagement intensity and therapeutic efficacy in obesity pharmacotherapy, with implications extending beyond traditional medication‐only approaches.

The principal strengths of our investigation include the substantial cohort size (*N* = 106 653), representing one of the largest real‐world analyses of dual GIP/GLP‐1RA and GLP‐1RA efficacy to date, and the use of MMRM modelling, which was tailored to account for variable follow‐up durations and the missing data patterns typical of observational research. The longitudinal design spanning 11 months enables robust characterisation of temporal weight loss dynamics, while comprehensive digital engagement stratification provides granular insights into technological augmentation of pharmacological efficacy. Studies comparing integrated pharmacological‐digital efficacy for digital weight loss services remain scarce with large population datasets. Our previous real‐world study was one of the largest cohort datasets currently for dual GIP/GLP‐1RA and GLP‐1RA, with a five‐month follow‐up analysis study showing engaged participants achieved 9.0% mean weight loss (95% CI 8.9%–9.1%) versus 5.9% (95% CI 5.8%–6.0%) in non‐engaged participants (*p* < 0.001).^11^

We acknowledge several limitations that may constrain causal inference. The retrospective design and non‐randomised allocation introduce the chance of potential selection bias and confounding, as digitally engaged participants may have unmeasured characteristics that predispose them to greater treatment adherence or capacity for lifestyle modification. Self‐reported weight measurements introduce human error, measurement bias and reporting bias, potentially complicating outcome ascertainment. Despite adjustment for baseline demographic and clinical variables such as age, sex, BMI, and comorbidities, residual unmeasured confounding from socioeconomic factors, health literacy, digital competency or intrinsic motivation remains possible. Considering this, future work for digital engagement and weight loss services should consider including propensity score matching or instrumental variable approaches.

While we captured baseline comorbidities, the absence of longitudinal biomarker data prevented assessment of whether digital engagement differentially impacts diabetes control (HbA1C trajectories), blood pressure normalization rates, or lipid panel improvements. Future studies incorporating routine clinical measurements would strengthen understanding of the metabolic benefits beyond weight loss.

The real‐world population in an open cohort setting exhibited varying follow‐up times, censoring, and discontinuation, evidenced by decreasing cohort sizes from months 0 through 11. However, these declining cohort sizes at later timepoints primarily reflect our open cohort design with continuous enrollment (August 2024 to July 2025) rather than uniformly high discontinuation rates. Patients enrolling later had shorter maximum possible follow‐up due to administrative censoring at study end. While this introduces variable follow‐up durations, it mirrors real‐world clinical practice and enhances external validity. Our survival analysis methods appropriately account for both censoring and discontinuation. Among those with adequate follow‐up opportunity, discontinuation reflected common real‐world patterns including target weight achievement, economic factors, and medication supply shortages affecting GLP‐1RA availability during 2024–2025. These findings strengthen generalisability to routine practice settings. This mirrors findings in dual GIP/GLP‐1RA and GLP‐1RA studies where high discontinuation rates are common within the first year. Rodriguez and colleagues demonstrated discontinuation rates up to 64.8% in >125 000 patients from US electronic health records.^22^ Our future studies will address discontinuation data as the dataset matures. The engagement classification, while comprehensive in encompassing coaching utilisation, app engagement, and self‐monitoring behaviours, represents a composite metric that may obscure differential effects of specific intervention components. Safety analysis revealed a paradoxical pattern whereby engaged participants exhibited higher absolute incident rates (RR 1.55, 95% CI: 1.11–2.16) yet demonstrated comparable or lower incidence rates when accounting for exposure time (IRR 0.83, 95% CI: 0.60–1.15). This likely reflects surveillance bias, with engaged participants demonstrating increased healthcare utilisation and adverse event reporting rather than genuine safety signal amplification. The predominance of low‐severity events (prescribing errors 40.0%, side effects 30.8%, majority being no harm or low harm) supports this interpretation, as safety information is only logged when highlighted to clinical or risk management teams, thus introducing reporting bias.

The efficacy of GLP‐1 receptor agonists in obesity treatment has been established through numerous large‐scale clinical trials. Wong et al.'s meta‐analysis of 47 RCTs with 23 244 GLP‐1 RA patients showed −4.57 kg (95% CI −5.35 to −3.78) weight reduction across 4–104 weeks with single GLP‐1 RA therapy.^23^ Our study exceeds this population by 4.5‐fold, highlighting the valuable lessons outcomes attainable from integrated digital health services. Landmark RCTs have demonstrated substantial efficacy: STEP 1 semaglutide 2.4 mg achieved −14.9% versus −2.4% placebo at 68 weeks, with 86.4% achieving ≥5% weight loss.^8^ The latest STEP UP trial results showed higher dose semaglutide 7.2 mg achieved between −18.7% and −20.7% weight loss at 72 weeks in adults with obesity (BMI ≥30 kg/m^2^) without type 2 diabetes.^24^ Whereas SURMOUNT‐5 showed tirzepatide superiority over semaglutide (−20.2% vs. −13.7% at 72 weeks) in 751 non‐diabetic participants.^6^ Our study's engaged participants achieved −21.5% (95% CI: −22.0, −21.1, *p* < 0.001) WL by month 11, extending RCT findings in routine practice.

Reassuringly, our findings are aligned with those from other real‐world evidence studies. Clark and colleagues reported 8.0% WL at month 12 in 66 094 virtual care participants, with 64.2% achieving ≥5% weight loss,^25^ while our engaged cohort achieved superior rates (79.4% vs. 36.3% for ≥5% weight loss). Second Nature's evaluation showed lower WL outcomes compared to our study with 19.1% WL (the mean weight loss was 20.0 kg; SD 8.7 kg; *p* < 0.001) at 12‐month follow‐up, though with withdrawal bias.^26^

Prescribing safety concerns in digital services were highlighted by Talay et al., who identified errors in 4.4% of 37 323 GLP‐1 RA prescriptions (error rate at 44.3 per 1000 prescriptions), predominantly insufficient safety counselling (49.15%) and inadequate contraindication investigations (30.29%),^27^ in contrast, our study demonstrated substantially lower prescribing error rates at 1.71 per 1000 patients, suggesting that integrated clinical oversight and systematic safety protocols can significantly reduce prescription‐related incidents in digital obesity care.

In our comprehensive safety analysis of the Voy DWLS, we captured 455 clinical incidents across 106 653 participants (4.15 per 1000 patients), with prescribing errors comprising 40% of incidents, providing surveillance data addressing digital healthcare quality concerns. Finally, Tanashat and colleagues' meta‐analysis of 19 RCTs with 32 884 non‐diabetic patients found GLP‐1 RA increased adverse events (RR 1.11; 95% CI 1.05–1.16) versus placebo.^28^ Engaged participants showed higher incident risk (RR 1.55; 95% CI 1.11–2.16) but lower incident rates per person‐month (IRR 0.83; 95% CI 0.60–1.15), suggesting enhanced surveillance rather than increased harm as a possible cause.

## 5 CONCLUSION

This analysis of over 100 000 adults demonstrates that digital engagement significantly enhances dual GIP/GLP‐1RA and GLP‐1RA outcomes, with engaged participants achieving superior weight loss (−21.5% vs. −17.0%), 42–86% faster milestone achievement across all thresholds (≥5% to ≥25% weight loss), and comparable safety profiles when accounting for exposure time. Future RCTs are essential to establish causality, while long‐term prospective studies examining real‐world sustainability with comprehensive metabolic profiling are needed to evaluate whether enhanced digital engagement translates to superior cardiometabolic outcomes beyond weight loss, with further exploration on discontinuation patterns, and cost‐effectiveness will inform clinical implementation for patient‐centred DWLS that are both safe and efficacious.

## AUTHOR CONTRIBUTIONS

All authors contributed to the conception and design of the study. Hans Johnson performed the data analysis and interpretation of the results, along with Ashley Kieran Clift (interpretation). Hans Johnson and Ashley Kieran Clift designed and ran (Hans Johnson) the statistical analysis, with the assessment of the analysis by Hans Johnson, Ashley Kieran Clift, Daniel Reisel, and David R. Huang. Hans Johnson drafted the manuscript, and all authors critically reviewed and approved the final version for publication.

## FUNDING INFORMATION

No external funding was received for this study. The research was conducted as part of routine service evaluation activities within the organisation with further ethics approval from University College London (UCL) Research Ethics Committee (REC) to conduct a cohort study analysis.

## CONFLICT OF INTEREST STATEMENT

The authors Hans Johnson, David R. Huang, Ashley Kieran Clift and Daniel Reisel are members within the organisation Voy, Menwell™; Hans Johnson is the clinical researcher, Ashley Kieran Clift is the head of clinical research, David R. Huang is the innovation director, and Daniel Reisel is the research advisor.

## Supporting information


**TABLE S1.** Weight loss trajectories for dual GIP/GLP‐1RA by engagement status. WL% = percentage weight loss from baseline (negative values indicate weight loss). Difference = engaged WL% − not engaged WL% (negative values favour engaged group for greater weight loss). *p*‐values derived from MMRM interaction terms (engagement × time). CI = 95% Confidence Interval; *N* = number of patients. All *p*‐values <0.001 indicate statistically significant differences between groups.
**TABLE S2.** Weight loss trajectories for GLP‐1RA by engagement status. WL% = percentage weight loss from baseline (negative values indicate weight loss). Difference = engaged WL% − not engaged WL% (negative values favour engaged group for greater weight loss). P‐values derived from MMRM interaction terms (engagement × time). CI = 95% Confidence Interval; *N* = number of patients. Statistical significance varies by timepoint due to smaller sample sizes.
**TABLE S3.** Clinical incident types in dual GIP/GLP‐1RA and GLP‐1RA cohort. Incident types ranked by frequency. Rates calculated per 1000 patients in the total dual GIP/GLP‐1RA and GLP‐1RA cohort (*N* = 106 653). CI = 95% Confidence Interval. Incident rates per 1000 patients with 95% confidence intervals were calculated using Poisson distribution methods.

**DATA S1.** STROBE Checklist.

## Data Availability

The datasets generated or analysed during this study are not publicly available due to the nature of the clinical data collected and the consent provided by patients; the individual participant data used in this study is not publicly available. The statistical code used in the analyses can be made available to researchers upon request to the corresponding author. Requests for access to anonymised data for non‐commercial projects should be directed to the corresponding author and will require appropriate ethical approvals but are available from the corresponding author on reasonable request.
