# Supplementary material for: Digital engagement enhances dual GIP/GLP‐1 receptor agonist and GLP‐1 receptor agonist efficacy: A retrospective cohort analysis of a digital weight loss service on outcomes and safety
Source: Diabetes Obes Metab. 2025 Oct 27;28(1):634–43. doi: 10.1111/dom.70244 (PMC12673462; doi:10.1111/dom.70244)
Supplement: Supplementary file 1 — TABLE S1. Weight loss trajectories for dual GIP/GLP‐1RA by engagement status. WL% = percentage weight loss from baseline (negative values indicate weight loss). Difference = engaged WL% − not engaged WL% (negative values favour engaged group for greater weight loss). p‐values derived from MMRM interaction terms (engagement × time). CI = 95% Confidence Interval; N = number of patients. All p‐values <0.001 indicate statistically significant differences between groups. TABLE S2. Weight loss trajectories for GLP‐1RA by engagement status. WL% = percentage weight loss from baseline (negative values indicate weight loss). Difference = engaged WL% − not engaged WL% (negative values favour engaged group for greater weight loss). P‐values derived from MMRM interaction terms (engagement × time). CI = 95% Confidence Interval; N = number of patients. Statistical significance varies by timepoint due to smaller sample sizes. TABLE S3. Clinical incident types in dual GIP/GLP‐1RA and GLP‐1RA cohort. Incident types ranked by frequency. Rates calculated per 1000 patients in the total dual GIP/GLP‐1RA and GLP‐1RA cohort (N = 106 653). CI = 95% Confidence Interval. Incident rates per 1000 patients with 95% confidence intervals were calculated using Poisson distribution methods. [file DOM-28-634-s002.docx]

**Supplementary material tables 1-3**

**Supplementary Table 1**. Weight loss trajectories for dual GIP/GLP-1RA by engagement status. WL% = percentage weight loss from baseline (negative values indicate weight loss). Difference = engaged WL% - not engaged WL% (negative values favor engaged group for greater weight loss). P-values derived from MMRM interaction terms (engagement X time). CI = 95% Confidence Interval; N = number of patients. All p-values <0.001 indicate statistically significant differences between groups.

| **Month** | **(N) Engaged** | **(N) Not engaged** | **Engaged WL% (95% CI)** | **Not engaged WL% (95% CI)** | **Mean WL difference** | **Relative WL difference %** | **P-value** |
| --- | --- | --- | --- | --- | --- | --- | --- |
| 0 | 5,967 | 95,682 | N/A | N/A | N/A | N/A | N/A |
| 1 | 5,730 | 44,789 | -4.0 (-4.1, -3.9) | -3.5 (-3.6, -3.5) | -0.5 | 14.3 | <0.001 |
| 2 | 5,081 | 33,522 | -7.2 (-7.4, -7.1) | -6.5 (-6.6, -6.4) | -0.8 | 12.3 | <0.001 |
| 3 | 4,010 | 24,428 | -10.0 (-10.1, -9.9) | -8.9 (-9.0, -8.8) | -1.1 | 12.4 | <0.001 |
| 4 | 2,838 | 16,856 | -12.4 (-12.6, -12.3) | -10.9 (-11.0, -10.8) | -1.5 | 13.8 | <0.001 |
| 5 | 1,908 | 12,156 | -14.7 (-14.8, -14.5) | -12.5 (-12.6, -12.4) | -2.2 | 17.6 | <0.001 |
| 6 | 1,253 | 9,190 | -16.5 (-16.7, -16.3) | -13.7 (-13.8, -13.6) | -2.8 | 20.4 | <0.001 |
| 7 | 826 | 5,836 | -18.0 (-18.2, -17.8) | -14.9 (-15.0, -14.8) | -3.1 | 20.8 | <0.001 |
| 8 | 522 | 3,831 | -19.3 (-19.6, -19.1) | -15.5 (-15.6, -15.4) | -3.8 | 24.5 | <0.001 |
| 9 | 377 | 2,642 | -20.3 (-20.6, -20.1) | -16.3 (-16.4, -16.1) | -4.1 | 25.2 | <0.001 |
| 10 | 234 | 1,486 | -21.5 (-21.8, -21.1) | -17.2 (-17.3, -17.0) | -4.3 | 25.0 | <0.001 |
| 11 | 114 | 748 | -22.0 (-22.5, -21.5) | -17.6 (-17.8, -17.4) | -4.4 | 25.0 | <0.001 |

# **______________________________________________________________________________**

# **Supplementary Table 2.** Weight loss trajectories for GLP-1RA by engagement status. WL% = percentage weight loss from baseline (negative values indicate weight loss). Difference = engaged WL% - not engaged WL% (negative values favor engaged group for greater weight loss). P-values derived from MMRM interaction terms (engagement X time). CI = 95% Confidence Interval; N = number of patients. Statistical significance varies by timepoint due to smaller sample sizes.

| **Month** | **(N) Engaged** | **(N) Not engaged** | **Engaged WL% (95% CI)** | **Not engaged WL% (95% CI)** | **Mean WL difference** | **Relative WL difference %** | **P-value** |
| --- | --- | --- | --- | --- | --- | --- | --- |
| 0 | 119 | 4,885 | N/A | N/A | N/A | N/A | N/A |
| 1 | 109 | 1,465 | -2.5 (-3.1, -2.0) | -2.1 (-2.4, -1.8) | -0.5 | 23.8 | 0.068 |
| 2 | 69 | 798 | -5.2 (-5.8, -4.5) | -4.4 (-4.7, -4.1) | -0.7 | 15.9 | 0.020 |
| 3 | 60 | 569 | -7.4 (-8.1, -6.7) | -6.4 (-6.8, -6.1) | -1.0 | 15.6 | 0.003 |
| 4 | 42 | 426 | -9.4 (-10.2, -8.6) | -8.1 (-8.4, -7.7) | -1.3 | 16.0 | 0.001 |
| 5 | 28 | 309 | -10.6 (-11.5, -9.6) | -9.3 (-9.7, -8.9) | -1.3 | 14.0 | 0.007 |
| 6 | 23 | 280 | -12.8 (-13.9, -11.8) | -10.0 (-10.4, -9.6) | -2.9 | 29.0 | <0.001 |
| 7 | 17 | 181 | -15.0 (-16.2, -13.9) | -10.7 (-11.2, -10.3) | -4.3 | 40.2 | <0.001 |
| 8 | 10 | 117 | -16.3 (-17.8, -14.9) | -11.5 (-12.0, -11.0) | -4.9 | 42.6 | <0.001 |
| 9 | 10 | 86 | -17.9 (-19.3, -16.4) | -12.8 (-13.4, -12.2) | -5.0 | 39.1 | <0.001 |
| 10 | 9 | 62 | -19.0 (-20.5, -17.4) | -13.1 (-13.7, -12.4) | -5.9 | 45.0 | <0.001 |
| 11 | 6 | 38 | -19.0 (-20.9, -17.1) | -12.4 (-13.2, -11.6) | -6.6 | 53.2 | <0.001 |

# **______________________________________________________________________________**

**Supplementary Table 3**. Clinical incident types in dual GIP/GLP-1RA and GLP-1RA cohort. Incident types ranked by frequency. Rates calculated per 1,000 patients in the total dual GIP/GLP-1RA and GLP-1RA cohort (N=106,653). CI = 95% Confidence Interval. Incident rates per 1,000 patients with 95% confidence intervals were calculated using Poisson distribution methods.

| **Category** | **Incident type** | **Count** | **Percentage of all clinical incidents** | **Rate per 1,000 patients (95% CI)** |
| --- | --- | --- | --- | --- |
| **Harm level** |  |  |  |  |
|  | Minor | 210 | 46.2% | 1.97 (1.71-2.25) |
|  | No Harm | 191 | 42.0% | 1.79 (1.55-2.06) |
|  | Near Miss | 43 | 9.5% | 0.40 (0.29-0.54) |
|  | Moderate | 9 | 2.0% | 0.08 (0.04-0.16) |
|  | Major | 2 | 0.40% | 0.02 (0.00-0.07) |
| **Incident** **type** |  |  |  |  |
|  | Prescribing error | 182 | 40.0% | 1.71 (1.47-1.97) |
|  | Side effects | 140 | 30.8% | 1.31 (1.10-1.55) |
|  | Other | 110 | 24.2% | 1.04 (0.80-1.18) |
|  | Inappropriate information | 16 | 3.5% | 0.15 (0.09-0.24) |
|  | Not specified | 5 | 1.1% | 0.05 (0.02-0.11) |
|  | Allergic reaction | 2 | 0.4% | 0.02 (0.00-0.07) |
