# Supplementary material for: Digital engagement enhances dual GIP/GLP‐1 receptor agonist and GLP‐1 receptor agonist efficacy: A retrospective cohort analysis of a digital weight loss service on outcomes and safety
Source: Diabetes Obes Metab. 2025 Oct 27;28(1):634–43. doi: 10.1111/dom.70244 (PMC12673462; doi:10.1111/dom.70244)
Supplement: Supplementary file 2 — DATA S1. STROBE Checklist. [file DOM-28-634-s001.docx]

Supplementary File 1 - STROBE Checklist

# Digital engagement enhances dual GIP/GLP-1 receptor agonist and GLP-1 receptor agonist efficacy: a retrospective cohort analysis of a digital weight loss service on outcomes and safety

## Detailed STROBE Checklist

| Item | STROBE Recommendation | Page | Where Addressed |
| --- | --- | --- | --- |
| 1a | Indicate the study's design in the title or abstract. | 1-2 | Title includes "retrospective cohort analysis"; Abstract Method section specifies design. (Title; Abstract lines 24-25) |
| 1b | Provide an informative, balanced abstract. | 1-2 | Abstract (250 words) summarises aim, methods (Aug 2024-Jul 2025, UKN=106,653), engagement definition, MMRM/KM analyses, key results with CIs, conclusions. (Abstract lines 20-44) |
| 2 | Explain scientific background and rationale. | 3-5 | Introduction covers obesity epidemic, GLP-1RA efficacy, digital health integration rationale, safety considerations, knowledge gaps. (Introduction lines 50-96) |
| 3 | State specific objectives/hypotheses. | 5 | Primary objective clearly stated: characterise real-world WL efficacy, quantify digital engagement impact, establish safety profile. (Introduction lines 98-101) |
| 4 | Present key elements of study design early. | 4 | Methods: UK-wide Voy DWLS, August 2024 to July 2025, open cohort enrollment, variable follow-up durations, data collection methods described. (Methods lines 104-113) |
| 5 | Describe setting/locations/dates. | 5 | Methods: UK-wide Voy DWLS, Aug 2024-Jul 2025, follow-up periods, data collection methods described. (Methods lines 104-122) |
| 6a | Eligibility criteria; sources/methods of selection. | 6 | Methods-Participants: adults ≥18 years, BMI ≥30 or ≥27 with comorbidity, dual GIP/GLP-1RA or GLP-RA monotherpay therapy via Voy platform. (Methods lines 115-119) |
| 6b | For matched studies, give matching criteria. | N/A | Not applicable-unmatched cohort study with covariate adjustment in statistical models. |
| 7 | Clearly define outcomes, exposures, predictors. | 6-7 | Methods-Outcomes: primary (% weight change), secondary (thresholds), safety outcomes defined. Digital engagement definition detailed. (Methods lines 129-144) |
| 8 | Data sources and assessment methods. | 6-7 | Methods: Voy app data sources (weight, height, BMI, comorbidities), quality assurance data for safety outcomes. (Methods lines 125-139) |
| 9 | Efforts to address potential bias. | 7-8 | Methods-Data processing: exclusion criteria for biologically improbable measurements to reduce bias. (Methods lines 157-163). |
| 10 | Explain how study size was arrived at. | 9 | Methods Statistical analysis subsection: power analysis determined minimum 118 participants per engagement group would provide 80% power to detect 15% difference in proportion achieving ≥10% weight loss at 5% significance level. Methods Lines 199-201 |
| 11 | Explain handling of quantitative variables. | 8-9 | Methods Statistical analysis subsection: percentage weight change defined as change from baseline (negative values indicate loss); continuous variables as means with standard deviations; categorical variables as counts with percentages; baseline adjustment variables specified; threshold categorisation detailed; baseline weight centered for correlation analyses (Methods lines 166-192). |
| 12a | Statistical methods, confounding control. | 8-9 | Methods: MMRM with fixed effects for engagement status, month, and their interaction; adjustment for baseline age, BMI, sex, and comorbidities (diabetes, high cholesterol, hypertension, PCOS, MASLD); compound symmetry covariance structure with Satterthwaite degrees of freedom; Kaplan-Meier methods with log-rank tests; Cox proportional hazards models for time-to-event analyses (Methods lines 169-192) |
| 12b | Methods for subgroups/interactions. | 8-9 | Methods: engagement×month interaction term in MMRM, engagement stratification in KM analysis. (Methods lines 164-181) |
| 12c | How missing data were addressed. | 8-9 | Methods: MMRM handles missing data under MAR assumption, no imputation performed. (Methods lines 170-172) |
| 12d | How loss to follow-up was addressed. | 6 | Methods: follow-up from first prescription until treatment cessation (discontinuation or target weight achievement) or cohort window end with administrative censoring; open cohort design with variable follow-up explicitly acknowledged; survival analysis methods appropriately account for censoring (Methods lines 118-120; Statistical analysis section lines 189-192) |
| 12e | Sensitivity analyses. | 8-9 | Methods mentions prespecified sensitivity fits with alternative covariance structures (Methods lines 168-170) |
| 13a | Numbers at each study stage. | 14 | Results: N=106,653 total, 84,955 used app, 6,086 engaged, 100,567 not engaged. Month-by-month participation in Table 2. (Results lines 270, Table 2) |
| 13b | Reasons for non-participation. | 21 | Results acknowledge decreasing cohort sizes over time, Discussion mentions discontinuation patterns typical of GLP-1RA studies. ( , Discussion lines 383-400) |
| 13c | Flow diagram. | 15 | Figure 1 shows weight loss trajectories and table 2 shows total numbers but no participant flow diagram provided. |
| 14a | Participant characteristics and exposures. | 11-12 | Results-Table 1: comprehensive baseline characteristics by engagement status (demographics, anthropometrics, comorbidities). (Table 1 lines 237-238) |
| 14b | Missing data for each variable. | 11 | Table 1 caption notes "Baseline age and BMI were fully recorded with no missing data." (Table 1 caption lines 232-236) |
| 14c | Summarise follow-up time. | 17 | Results: 290,050 person-months total follow-up, 11-month analysis timeframe. (Results lines 308-312) |
| 15 | Outcome events/summary measures over time. | 11-18 | Results: Table 2 (weight loss trajectories), Table 3 (milestone achievement), Table 4 (safety outcomes), Figure 1 (trajectories). (Results) |
| 16a | Adjusted estimates with precision and confounders. | 14-15 | Results: MMRM-adjusted estimates with 95% CIs reported, covariates specified (age, BMI, sex, comorbidities). (Results Table 2, lines 270-280) |
| 16b | Category boundaries for continuous variables. | 6-8,16-17 | Methods: weight loss thresholds (≥5%, ≥10%, ≥15%, ≥20%, ≥25%) clearly defined. (Methods lines 187-192, Results Table 3) |
| 16c | Translate relative risk to absolute risk. | 14-16 | Results: absolute percentage point differences reported alongside relative measures, achievement percentages provided. (Results Table 2-3) |
| 17 | Other analyses (subgroups, interactions). | 14-17 | Results: engagement×time interactions tested throughout 11 months with p-values; medication-specific subgroup analyses (dual GIP/GLP-1 RA: -22.0% vs -17.6%, 4.4 percentage point difference; monotherapy GLP-1 RA: -19.0% vs -12.4%, 6.6 percentage point difference); safety analyses stratified by engagement with multiple effect measures; weight regain analysis (1.1% vs 3.1%); baseline weight correlation analysis by engagement group (r=-0.21 vs r=-0.11) (Results lines 254-263 for medication subgroups, 290-305 for weight regain and baseline weight, 308-326 for safety) |
| 18 | Summarise key results with reference to objectives. | 18 | Discussion-Summary: key findings related to stated objectives (efficacy, engagement impact, safety profile). Lines 334-conclusion |
| 19 | Discuss limitations and potential bias. | 20-21 | Discussion-Strengths and weaknesses: retrospective design, selection bias, self-reported weights, residual confounding extensively discussed. (Discussion lines 366-408) |
| 20 | Cautious interpretation considering limitations. | 18-23 | Discussion: balanced interpretation acknowledging limitations, comparison with literature, surveillance bias explanation. (Discussion lines) |
| 21 | Discuss generalisability. | 23 | Discussion to Conclusion: mentions implementation for patient-centred DWLS (Discussion lines 447-455) |
| 22 | Funding source and role. | 25 | Funding: "No external funding was received...routine service evaluation activities...ethics approval from UCL." (Funding lines 473-475 |
